# A Guide to Periprocedural Skin Care Regimen for Injectable and Nonenergy Cosmetic Procedures Based on a Consensus of 6 Aesthetic Practitioners

**DOI:** 10.1093/asjof/ojaf121

**Published:** 2025-09-30

**Authors:** Cara McDonald, Leona Yip, John Sullivan, Wenyuan Liu, Frank Lin, Greg Goodman

## Abstract

The use of injectable aesthetic treatments (including fillers, neurotoxins, polynucleotides, and biostimulators) and nonenergy surface-active aesthetic treatments (such as chemical peels, microneedling, and microdermabrasion) is increasing. To date, little guidance is available in the literature concerning periprocedural skincare for these aesthetic procedures. The aim of the authors of this study is to provide periprocedural skincare recommendations and an algorithm to guide holistic skincare that will enhance and retain procedural effects and optimize skin health in the longer term. A panel of 6 Australian aesthetic practitioners (5 dermatologists and 1 plastic surgeon) developed generalized periprocedural skincare recommendations for injectable and surface-active aesthetic treatments. Recommendations were stratified into 2 categories: skin barrier nondisruptive procedures (fillers, threads, neuromodulators, and polynucleotides) and skin barrier disruptive procedures (nonenergy surface-active procedures). Establishing a good preprocedural basic skincare routine (cleanser, moisturizer, and broad-spectrum sun protection factor 50+ sunscreen) 2 to 4 weeks before a procedure is recommended. Actives including vitamin A, vitamin B3, antioxidants, hyaluronic acid, and lipids may also be advised and tailored to an individual's skin condition. It is suggested that toners and exfoliants can be avoided, whereas antioxidants, tranexamic acid, and growth factors can be used immediately following needling procedures. Hyaluronic acid and antioxidants may be used immediately following chemical peels along with bland skincare. Postprocedural makeup and skincare actives should be avoided immediately post surface-active procedures. The implementation of periprocedural skincare regimens may improve treatment-related outcomes and reduce recovery time. Furthermore, frequency and severity of potential side effects may be reduced.

**Level of Evidence:** 5 (Therapeutic)

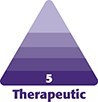

There is a continued global rise in the demand and use of aesthetic facial rejuvenation treatments such as fillers, neurotoxins, polynucleotides, biostimulators, chemical peels, microneedling, and microdermabrasion.^1-3^ This trend is driven by factors such as increased demand for minimally invasive procedures, a cultural shift toward acceptance of aesthetic treatments, and advancements in technology.^2,4^

Although considered relatively safe, injectable and nonenergy surface-active aesthetic treatments can result in adverse events. To date, little guidance is available in the literature concerning periprocedural skincare for such aesthetic procedures.^5,6^ The aim of the authors of this review is to provide periprocedural recommendations (Figure 1) and an algorithm to guide the use of cosmeceuticals to improve treatment outcomes and reduce the likelihood adverse events. These recommendations also aim to optimize the skin condition before treatment and to complement and enhance postprocedure results where possible. The recommendations are based on the opinion of a group of experienced Australian clinicians (5 dermatologists and 1 plastic surgeon) and are classed as Level V evidence (consensus). Recommendations were developed at a face-to-face workshop. Disagreements were resolved through discussion, and 100% consensus was reached on all matters. The panel experts were present in a personal capacity and did not represent any professional body.

**Figure 1.**
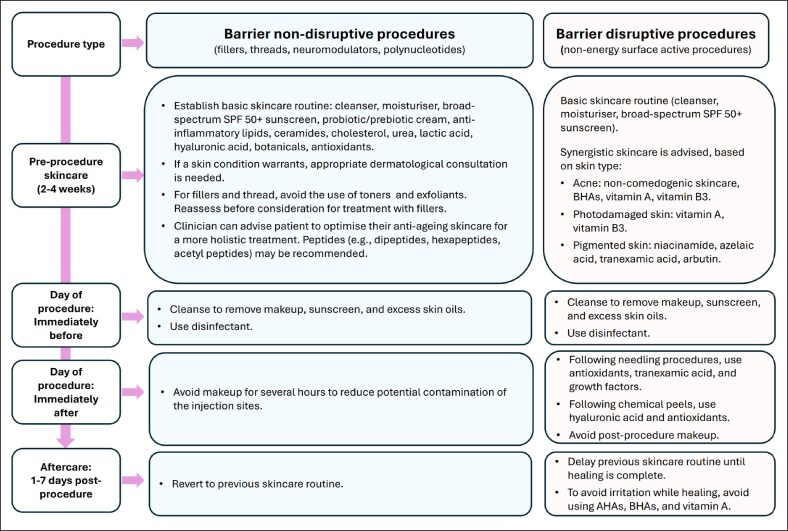
Algorithm for periprocedural skincare for facial injectable and nonenergy dermatologic procedures. Recommendations have been stratified into 2 categories: skin barrier nondisruptive procedures (fillers, threads, neuromodulators, and polynucleotides) and skin barrier disruptive procedures (nonenergy surface-active procedures). AHA, alpha-hydroxy acids; BHA, beta-hydroxy acids.

The presented recommendations are stratified into 2 categories: skin barrier nondisruptive procedures (fillers, threads, neuromodulators, and polynucleotides) and skin barrier disruptive procedures (nonenergy surface-active procedures). In addition to the presented recommendations, we recommend that skin dermatoses be examined by a qualified medical practitioner before any aesthetic procedure.

## BARRIER NONDISRUPTIVE PROCEDURES

### Neuromodulators and Polynucleotides

Cosmetic neuromodulators are injections of botulinum toxin that are utilized to treat a variety of cosmetic indications, including dynamic wrinkles, such as glabella lines and crow's feet, facial contouring, and skin quality improvements. They have become one of the most widely used aesthetic treatments.^7^ Polynucleotides are used to improve skin elasticity and hydration, although more research is needed to fully understand their mechanisms of action and optimal use.^8^

### Fillers and Threads

A sign of aging is the loss of volume in the subcutaneous fat compartments of the face. Facial fillers have become commonplace in the cosmetic industry, used to counteract age-related facial volume loss. There are multiple types of injectable fillers, including hyaluronic acid, biostimulators such as poly-L-lactic acid, calcium hydroxyapatite, hybrid, and other emerging products.^9^

Another sign of aging is the loosening and sagging of facial skin. A thread lift, or threads, is procedure, wherein temporary sutures are used to produce a subtle but visible contouring in the skin. These procedures require differentiation from injection of neurotoxins and polynucleotides because of the sustained foreign body postprocedure and increased risks associated with this.

### Preprocedure Skincare for Procedures That are Nondisruptive to the Skin Barrier

A basic skincare routine comprised of an appropriate cleanser, moisturizer, and broad-spectrum sun protection factor (SPF) 50+ sunscreen should be commenced 2 to 4 weeks before treatment in order to optimize the skin barrier. The utilization of appropriate moisturizers, gentle cleansers, and sunscreens should be recommended with consideration given to appropriate active antiaging ingredients, such as retinoids, peptides, antioxidants, and skin acids.

Products containing anti-inflammatory lipids, cholesterol, urea, lactic acid, hyaluronic acid, botanicals, probiotics/prebiotics, and antioxidants can be used. The utilization of such actives supports skin barrier function, thus optimizing skin health. The topical use of lipids, such as ceramides and cholesterol, has been shown to prevent water loss, improve skin hydration, and enrich the intercellular lipid-enriched matrix of the stratum corneum.^10,11^ Urea, glycerin, and hyaluronic acid act as humectants, increasing water content in the stratum corneum.^12^ Some botanicals have been shown to have anti-inflammatory, antimicrobial, and antioxidant properties, thus improving skin quality and supporting skin barrier function.^13^ For example, plant oils, such as jojoba and avocado oil, form a film on the skin surface to maintain moisture, enhance barrier repair, and reduce water loss from the skin.^14^ Cucumber extract is rich in vitamin A and vitamin C, whereas aloe vera is rich in magnesium lactate, salicylic acid, and polysaccharides, and these botanicals can soothe and heal irritated skin.^15-17^ Although many botanicals contain soothing and anti-inflammatory properties, their efficacy is not consistently supported by high-level clinical data and will likely depend greatly on the formulation.

Alcohol-based toners and physical exfoliants should be avoided in the weeks leading up to these procedures because they can dry the skin and potentially cause skin barrier disruption and skin irritation. People undergoing chemical peel treatment for melasma may benefit from priming with hydroquinone, because it has been shown to minimize the risk of postinflammatory hyperpigmentation and increase the likelihood of continued improvement postprocedure.^18-20^ Priming with retinoids can promote a more uniform peel penetration; however, these are normally discontinued 7 days before the procedure to avoid deeper penetration of peels into the dermis.^18^

On the day of the procedure, the skin should be cleaned using appropriate cleansers able to remove makeup, sunscreen, and excess skin oils. The use of a disinfectant on the full treatment field, such as aqueous chlorhexidine, povidone iodine, or hypochlorous acid, is needed to reduce the chance of contamination and microbial infections. It is recommended that this be followed by alcohol swabbing of injection sites. Active infection at the injection site is one of the contraindications for neuromodulators, and the treatment area should be examined before treatment administration.^7^

### Postprocedure Skincare for Procedures That are Nondisruptive to the Skin Barrier

No particular posttreatment skincare is needed after neuromodulators, polynucleotides, fillers, and threads, because the skin barrier is not disrupted by these procedures. Patients can resume their usual skincare routine the day after the procedure; however, care should be taken to avoid pressure and rubbing on the immediate treatment area. If bruising occurs, a number of therapies are available to limit its duration. Although there is no high-level evidence supporting or refuting the recommendation to avoid makeup after neuromodulator or dermal filler treatment to reduce the risk of complications, the prevailing expert consensus is to minimize any potential contamination of injection sites in the immediate postprocedure period and avoid makeup for at least several hours postinjection as a precautionary measure.^21^ An antiaging skincare routine should be recommended based on skin type and skin conditions to complement the aesthetic impact of the cosmetic procedure.

## BARRIER DISRUPTIVE PROCEDURES

### Nonenergy Surface-Active Procedures

Surface-active procedures may involve energy-based devices to induce an alteration in the surface layers of the skin to effect positive change. However, there is a rise in nonenergy-based technologies, such as peels, microneedling, microdermabrasion, microblading, and extractions, which impact the skin surface without energy being imparted to the skin. Microneedling is a skin rejuvenation technique that stimulates the wound-healing cascade and promotes new collagen deposition without significant damage to the overlying epidermis. Chemical peels act to remove skin layers from the surface of the skin in order to rejuvenate the skin and reduce fine lines, pigmentation, and texture. The depth of the chemical peel (epidermal vs dermal) can vary based on the chemical agent used. Microdermabrasion is a manual exfoliation of the epidermis to remove dead skin cells, debris, and impurities, thus improving skin appearance.

### Preprocedure Skincare for Procedures That are Disruptive to the Skin Barrier

A basic skincare routine comprised of an appropriate cleanser, moisturizer, and broad-spectrum SPF 50+ sunscreen should be commenced 2 to 4 weeks before treatment to optimize skin condition. Probiotic/prebiotic creams, lipids, ceramides, cholesterol, urea, lactic acid, hyaluronic acid, botanicals, and antioxidants are recommended in the preprocedure period as ingredients that support skin barrier function and hydration. Excessive skin irritants, such as alcohol-based toners and exfoliants, should be avoided. In addition to these basic skincare requirements, synergistic skincare tailored to specific skin conditions or skin types is advised. People with acne or acne-prone skin should be advised to utilize noncomedogenic products to avoid the formation of acne lesions and to reduce skin irritation before surface-active procedures.^22^ The use of water-based or oil-free sunscreen is advisable.^23^ Beta-hydroxy acids (BHAs), such as salicylic acid, are commonly utilized to treat acne; salicylic acid can help dissolve intercellular lipids, lyse comedones, reduce inflammation, and improve the appearance of skin.^24^ Vitamin B3, also known as nicotinamide, can reduce acne severity, the production of sebum, and skin appearance.^25-29^ People with photodamaged skin are advised to use vitamin A and vitamin B3 because these compounds have been shown to promote the repair of photodamaged skin.^30,31^ The use of niacinamide, azelaic acid, and arbutin is recommended for pigmented skin because they have been shown to reduce dark spots, lighten skin tone, and enhance the skin's overall texture and clarity.^32,33^

On the day of the procedure, the skin should be cleaned using appropriate cleansers able to remove makeup, sunscreen, and excess skin oils. This should be followed by the use of a disinfectant, which is needed to reduce the chance of infections. Aseptic practice can be variable, and minimum standards for asepsis must be met; good aseptic practice has been outlined by Murthy et al.^21^

### Postprocedure Skincare for Procedures That are Disruptive to the Skin Barrier

Makeup should not be used immediately following surface-active procedures. Individuals may be advised to use topical antioxidants, tranexamic acid, and growth factors immediately following needling procedures because these products have been shown to enhance the regenerative process of needling-induced wound healing. These compounds have also been reported to enhance the beneficial effects of needling procedures for skin rejuvenation. Although less high-level evidence and clinical data exist for tranexamic acid as a topical ingredient, a meta-analysis found tranexamic acid to significantly enhance the effects of microneedling for the treatment of melasma.^34^ The use of growth factors has also been reported to have an additive effect in improving skin following microneedling.^35^ After chemical peeling, it is commonplace and reasonable to use antioxidants to promote wound healing, as well as hydrating substances such as hyaluronic acid, aloe, and calming botanical agents.

In the week postprocedure, it is advised that makeup and the previous skincare routine are delayed until skin healing is complete. To avoid irritation while healing, products containing alpha-hydroxy acids, BHAs, and vitamin A should be avoided. The disruption in the epidermis may facilitate the enhanced delivery or penetration of agents into the dermis for the following few days after the procedure. Thought should be given to the skin problem being addressed, and product choice should be tailored accordingly. Varying levels of evidence support agents, such as tranexamic acid, azelaic acid, licorice extracts, and niacinamide for pigment issues, and collagen-stimulating substances, such as retinoids, Bakuchiol, and hydroxy acids; *Centella asiatica* may be helpful in optimizing results in rejuvenation and atrophic scarring.

## GENERAL SKINCARE RECOMMENDATIONS

A daily skincare regimen that supports the skin barrier and improves skin appearance is recommended. At a minimum, this should include daily use of a cleanser, sunscreen, and moisturizer appropriate for the skin type. Tailored skincare should be considered for patients with dermatologic comorbidities, such as dermatitis, acne, or rosacea, in the pre- and postprocedure setting, with special consideration for increased risk of complications or exacerbation of the underlying skin condition.

High solar ultraviolet (UV) radiation exposure can accelerate facial aging because it promotes the development of fine and coarse wrinkles, irregular pigmentation, lentigines, a yellowish complexion, and a leathery, rough skin texture. Photoaging is primarily caused by the destruction of skin collagen because of various UV-induced biochemical and DNA disruptions.^36^ People in certain regions, such as Australia, are exposed to higher solar UV radiation levels than those who live in temperate northern countries, leading to increased photodamage and accelerated skin aging.^37^ For example, fair-skinned Caucasian and Asian women in Australia exhibit more severe signs of facial aging (facial lines and facial volume loss) up to 20 years earlier than those living in the United States, United Kingdom, and Canada.^37^ This is accelerated skin aging likely because of, in part, higher cumulative sun exposure because of the Australian outdoor and coastal lifestyle. As such, daily chemical and physical sun protection in regions with high UV radiation levels is highly recommended all year round to avoid the deleterious effects of UV radiation on skin aesthetics and to reduce the risk of skin cancer. Sun protection also plays a role in maintaining the results of some cosmetic treatments, such as chemical peels and microneedling, which aim to improve the cosmetic appearance of chronically sun-damaged skin. Hence, people undergoing aesthetic procedures should be advised to implement a good basic skincare routine that includes sun protection for a holistic antiaging approach that helps to maintain procedural results.

Measures advised for photoprotection include avoiding direct sun exposure at the time of day when UV ray intensity is high, the use of protective clothing and broadbrim hats, and the daily application of an appropriate sunscreen. Sunscreens act to absorb and/or reflect UV rays. The choice of sunscreen should be based on the level of UV protection it provides and the person's skin type. A “broad-spectrum” sunscreen with an SPF of 50+ is recommended at a minimum because broad-spectrum sunscreens filter both UVA and UVB light. Sunscreens are available in a wide variety of formulations; hence, the right formulation can be tailored to skin phototype and dermatoses (Table 1). The utilization of lighter formulation organic sunscreens with a water or light liquid base (eg, sprays) is recommended for acne skin because these have a nongreasy texture and are less likely to be comedogenic. UV radiation and heat can trigger or exacerbate erythema and telangiectasia in people with rosacea; hence, sunscreens containing dimethicone and/or cyclomethicone are recommended to avoid facial irritation and help support the skin barrier. Heavy occlusive sunscreens should be avoided in people prone to rosacea to avoid skin heating. People with melasma should opt for a tinted sunscreen with added iron oxide to block both UV radiation and visible light.^38^ The effectiveness of sunscreen is often compromised by insufficient application in real-world settings. The recommended standard application amount of 2 mg/cm^2^ corresponds to a total of 35 mL (∼7 teaspoons) for a full body application on an adult.^39^

**Table 1.** Sunscreen Formulation Recommendations for Various Dermatoses

Skin condition	Sunscreen formulation recommendations
Acne (or acne-prone skin)	Lighter formulations such as spray or lotion
Rosacea	Chemical or mineral sunscreen (avoid heavy occlusive sunscreens)
Sensitive skin	Physical or mineral sunscreen
Melasma	Tinted sunscreen with iron oxide

## LIMITATIONS

The recommendations in this article are based on the expert opinion of experienced specialists and may be subject to limitations, including potential biases and the possibility of collective ignorance.

## CONCLUSIONS

Injectable agents, whether they be neuromodulators, exosomes and polynucleotides, or fillers, not only benefit the patient but also provide a touchpoint for the suggestion of holistic skin care. The patient needs to enhance and retain their improvement and carry this forward to allow them to optimize their skin health in the longer term.

Although energy-based devices such as lasers, intense pulsed light and light-emitting diode, radiofrequency, photodynamic therapy, fractionated heat, and plasma are very useful in treating a variety of skin conditions, nonenergy-based treatments, such as chemical peels, microdermabrasion, microneedling, and others, can exert beneficial effects on the skin and provide treatments that may be more rapidly healing than their energy-based counterparts. These treatments may be improved by preprocedural and postprocedural skin care and again provide an opportunity to help the patient's skin throughout their future.
